# Cerebral venous sinus thrombosis (CVST) associated with SARS-CoV-2 vaccines: clues for an immunopathogenesis common to CVST observed in COVID-19

**DOI:** 10.1186/s44158-021-00020-9

**Published:** 2021-11-18

**Authors:** Anna Teresa Mazzeo, Alberto Noto, Alessio Asmundo, Francesca Granata, Karol Galletta, Raffaella Mallamace, Cesare De Gregorio, Francesco Puliatti, Maria Carolina Fazio, Antonino Germano’, Caterina Musolino, Guido Ferlazzo

**Affiliations:** 1 Division of Anesthesia and Critical Care. Department of Human Pathology G. Barresi, University of Messina, Messina, Italy; 2 Section of Legal Medicine, Department of Biomedical and Dental Sciences and Morphofunctional Imaging, University of Messina, Messina, Italy; 3 Neuroradiology Unit, Department of Biomedical, Dental Science and Morphological and Functional Images, University of Messina, Messina, Italy; 4 Cardiology Unit, Department of Clinical and Experimental Medicine, University of Messina, Messina, Italy; 5 Transplant Coordination, A.O.U. Policlinico “G. Martino”, Messina, Italy; 6 U.O.S.D Stroke Unit. D.A.I. Emergenze Tempo Dipendenti. A.O.U. Policlinico “G. Martino”, Messina, Italy; 7 Division of Neurosurgery, BIOMORF Department, University of Messina, Messina, Italy; 8 Division of Haematology, Department of Human Pathology G.Barresi, University of Messina, Messina, Italy; 9 Laboratory of Immunology and Biotherapy, Department of Human Pathology G.Barresi and Cell Factory UniMe Research Center, Division of Clinical Pathology, A.O.U. Policlinico “G. Martino”, University of Messina, Messina, Italy

**Keywords:** Cerebral venous sinus thrombosis, COVID-19, SARS-CoV2, Thrombosis, Thrombocytopenia, Vaccine, VITT, Brain death, Organ donation

## Abstract

Severe acute respiratory syndrome coronavirus type 2 has been responsible for an unprecedented pandemic, and nowadays, several vaccines proved to be effective and safe, representing the only available strategy to stop the pandemic. While millions of people have safely received vaccine, rare and unusual thrombotic events have been reported and are undergoing investigations to elucidate their nature. Understanding initial trigger, underlying pathophysiology and the reasons for specific site localization of thrombotic events are a matter of debate.

We here propose that rare cases of cerebral venous sinus thrombosis, a clinical event that may rapidly evolve to brain death, reported after COVID-19 vaccine, might be consequent to an immune response resulting in inflamed/damaged endothelium, an event similar to that described for cases of cerebral venous sinus thrombosis reported during COVID-19 and not necessarily related to anti-Platelets Factor 4 antibodies, as recently described. Remarkably, in the two patients presenting at our hospital with cerebral venous sinus thrombosis and evolved to brain death, proper tissue perfusion and function maintenance allowed organ donation despite extensive thrombosis in the organ donors, with favorable outcome at 6 months.

Increased vigilance, close multidisciplinary collaboration, and further prospective research will help to better elucidate a very rare and still not fully understood pathophysiological event associated with vaccines for severe acute respiratory syndrome coronavirus 2.

Severe acute respiratory syndrome coronavirus type 2 (SARS-CoV-2) has been responsible for an unprecedented pandemic since its first description, late 2019. Less than 1 year since the first appearance of the virus on the scene, several vaccines proved to be effective and safe and received approval for emergency use. Vaccines are essential to mitigate the effects of the SARS-CoV2 virus on public health, and their importance is universally recognized as the only available strategy to stop the pandemic.

While millions of people have safely received vaccine, rare and unusual thrombotic events, termed vaccine–induced immune thrombotic thrombocytopenia (VITT), have been reported [1–11], with a mortality of 39–41%, mainly for hemorrhagic or ischemic brain injury [6, 12]. Thrombotic events have been mainly associated with adenoviral vector-based vaccines, but more rarely described also in association with mRNA vaccines [13–15].

Although VITT is an extremely rare complication, it might be fatal, especially when presenting as cerebral venous sinus thrombosis (CVST), and this has created diffuse concerns and stimulated research to elucidate underlying pathomechanisms.

In this article, we propose our viewpoint on the mechanisms underlying CVST associated with COVID-19 vaccines, a partially unknown clinical event that may rapidly evolve to brain death, and we briefly report two fatal cases of ChAdOx1 nCov-19 (Astrazeneca) vaccine-associated CVST and thrombocytopenia occurred at our institution.

VITT has been recently reported to occur 5 to 48 days, median 14, after anti-COVID-19 vaccination, with cerebral veins being the most common thrombotic site at presentation, followed by the deep veins of the legs, pulmonary arteries, and splanchnic-vein thrombosis [16]. The baseline platelet count and the presence of intracranial hemorrhage are independent predictors of death, with an observed mortality of 73% among patients with platelet counts below 30,000 per cubic millimeter and intracranial hemorrhage [16].

However, CVST has also been reported as a rare neurologic complication of COVID-19 [17–25]. Very importantly, CVST is rare in the general population as well as after adenovirus-based SARS-CoV-2 vaccination, but appears to be several-fold more common in hospitalized patients with COVID-19, with a reported weighted average of 2.4, 3.6 and 207.1 per million, respectively [23]. Similarly, in a large population of 537,913 COVID-19 patients, the absolute incidence of CVT was 42.8 per million patients and was significantly greater than in a matched control cohort [24]. In a retrospective multicenter study of 13,500 consecutive patients with COVID-19 hospitalized in the USA, CVST was reported with an incidence of 8.8 per 10,000 during a 3-month study, extremely higher than the expected incidence in the general population of 5 per million annually [25].

CVST associated with COVID-19 should represent the result of a prothrombotic inflammatory state caused by anti-SARS-CoV2 immune response targeting vessel endothelium, which express ACE2, i.e., one of the main cellular receptors for SARS-CoV2. Indeed, endothelial injury and microvascular inflammation are recognized as a feature of COVID-19 and a major contributor to hypercoagulability observed along the disease.

Because all vaccines currently approved in our country act via the production of virus-associated proteins able to target angiotensin-converting enzyme 2 (ACE2), also the immune response occurring after vaccine could putatively generate inflammatory events targeting vascular beds, similarly to COVID-19.

Nevertheless, clinical presentation of the recently published cases of VITT has been proposed to resemble heparin-induced thrombocytopenia (HIT), a life-threatening complication of heparin treatment. HIT is caused by the production of platelet-activating IgG antibodies that recognize multimolecular complexes of (cationic) platelet factor 4 (PF4) bound to (polyanionic) heparin [26].

Clinical symptoms and laboratory features of this prothrombotic state have been described also in a subset of patients not having received heparin, referred as autoimmune HIT (aHIT) [26]. Indeed, besides heparin, other polyanions, such as DNA and RNA, polyphosphates and bacterial wall can induce the conformational changes in PF4 required to expose neo-antigens [26].

While most of the published reports hypothesized pathologic antibodies to PF4 as the keystone of VITT, similar to autoimmune HIT, we hypothesize that rare cases of CVST reported after vaccine might be also contributed by a prothrombotic event secondary to inflamed/damaged endothelium, similarly to what described for cases of CVST reported during COVID-19 and, thus, not necessarily related to anti-PF4 antibodies. The presence of anti-PF4 antibodies, which is detectable in 90% of vaccine-induced CVST patients [16], might be a concomitant phenomenon secondary to immune-mediated cell damage leading to circulating DNA, another negatively charged compound that forms an immunogenic complex with the positively charged PF4. As mentioned above, nucleic acids are able to bind PF4 and change its conformation, eventually resulting in exposure of new epitopes responsible of auto-antibodies production [27]. Interestingly, an 11-month follow-up study demonstrated that anti-PF4 antibodies are transient in most patients with VITT and become negative in 66% of the patients [28]. Furthermore, the kinetics of anti-PF4 antibodies in VITT compared to HIT is currently unknown and requires further studies [29]. Molecular mimicry between spike protein and PF4 epitopes has also been proposed [30]. More importantly, following immunization with COVID-19 vaccine, CVST can occur in the absence of anti-PF4 antibodies [31].

Considering the timing of CVST occurrence upon vaccine administration, a role for vaccine-induced, anti-spike immune response appears likely. We envisage, as a hypothesis for VITT pathogenesis, that the vaccine could lead to an adaptive immune response able to eventually target and damage cells that express the receptors for the spike-associated proteins induced by the vaccine, including endothelial cells. In this instance, a main pathogenic role would be played by the presence of not yet identified individual predisposing factors, which call for further research to better elucidate the mechanism and guide future directions.

The reasons why this immunogenic thrombosis preferentially occurs in cerebral venous vessels are still unclear, but a different density or polymorphism of specific immunoglobulin receptors (CD32) in endothelial cells might play a role [32]. Others suggest a strong expression of Coxsackie-adenovirus-receptors and adhesion molecules on central nervous system [33].

The endothelium contributes to the local balance between pro- and anti-inflammatory mediators as well as procoagulant and anticoagulant activities. Furthermore, in different vascular beds, endothelial cells exhibit properties that are specific of the local environment. This could further explain the different susceptibility of CVST as preferential site of VITT phenomena. Immunohistochemical findings of post-mortem examinations suggest endothelial activation sustaining procoagulant conditions and thrombus formation [34].

Given the physiological circulatory route of cerebrospinal fluid, thrombosis might occur due to the spread of vaccine-associated inflammatory mediators or effector cells to the vascular endothelium of the sinus venous or to direct extension into the sinus venous of the immunocomplexes present into the cerebrospinal fluid. Interestingly, SARS-CoV2 infection preceded the symptoms of CVST by up to two weeks, as reported in recent cases of VITT, which, again, represent the interval of time necessary for obtaining an effective adaptive immune response.

In Table 1 and Fig. 1, we present two fatal cases of ChAdOx1 nCov-19 (Astrazeneca) vaccine-associated CVST and thrombocytopenia, both complicated by cerebral hemorrhage rapidly evolving to brain death, that were admitted to our university hospital in March 2021. Worthwhile to signal, and in agreement with an immune-based pathogenesis associated with an endothelial injury, a high ScvO2 value occurred in case 2, which might therefore suggest an inability of the cells to extract oxygen or a micro-circulatory shunting due to endothelium damage.

**Table 1:** Characteristics and clinical course of two cases of cerebral venous sinus thrombosis post vaccination with ChAdOx1 nCoV-19 presenting at our University hospital and evolving to brain death

	Patient 1	Patient 2
Age and sex	55-years-old woman	45-year-old man
Preexisting conditions and screening for thrombophilia	No preexisting conditions. MTHFR A1298C homozygous mutation	Body mass index of 30 Screening negative
Symptoms and timing since vaccine administration	Low-grade fever and headache developed one week after vaccination	History of headaches 23 days after vaccination
Admission clinical picture, platelet count, and D-dimer (reference value 0–0.5ug/ml)	Alert and cooperative, headache Platelet count: 31,000 per cubic millimeter D-dimer > 4ug/ml	Acute focal neurologic motor deficit and altered level of consciousness Platelet count: 125,000 per cubic millimeter D-dimer > 4ug/ml
Sites of thrombosis during clinical course	Pulmonary subsegmental arteries thrombosis Thrombosis of the right internal jugular vein, absent signal void at straight sinus and at right transverse sinus (Fig. 1A–D) Portal vein branch thrombosis and partial thrombosis of inferior cava vein. Extension of thrombosis at mesenteric superior vein, main branches of portal vein, and sovraepatic branches	Massive thrombosis of the right transverse-sigmoid and of superior sagittal sinuses associated with a right intracerebral hemorrhagic infarction and midline shift (Fig. 1E–H) No signs of thrombosis were found in any other distric.
Therapy and interventions	Methylprednisolone, fondaparinux, intravenous immunoglobulins	Dexamethasone, fondaparinux, intravenous immunoglobulins
Antibodies to platelet factor 4, reference value < 0.400 OD	1.126 OD	2.37 OD
Evolution	Neurological deteriorations on day 2, pupils became anisocoric, Glasgow Coma Scale was 3 Cerebellar hemorrhage and diffuse cerebral edema with transtentorial herniation Emergency posterior decompressive craniectomy, intracranial pressure monitoring, external ventricular drainage. Refractory intracranial hypertension Death by neurologic criteria was declared on day 8	Pupils were dilated and fixed and Glasgow Coma Scale was 3. Neurosurgical consult excluded further intervention for futility due to poor grade prognosis Central venous oxygen saturation was 92%, without clinical signs of sepsis Death by neurologic criteria was declared on day 3
Organ donation and transplanted organs	Organ donation per previously expressed wish to donate Kidneys and corneas transplanted	Organ donation per previously expressed wish to donate Lungs, liver, kidneys. and corneas transplanted
Outcome in organ recipients	Good at 6 months	Good at 6 months

**Fig. 1:**
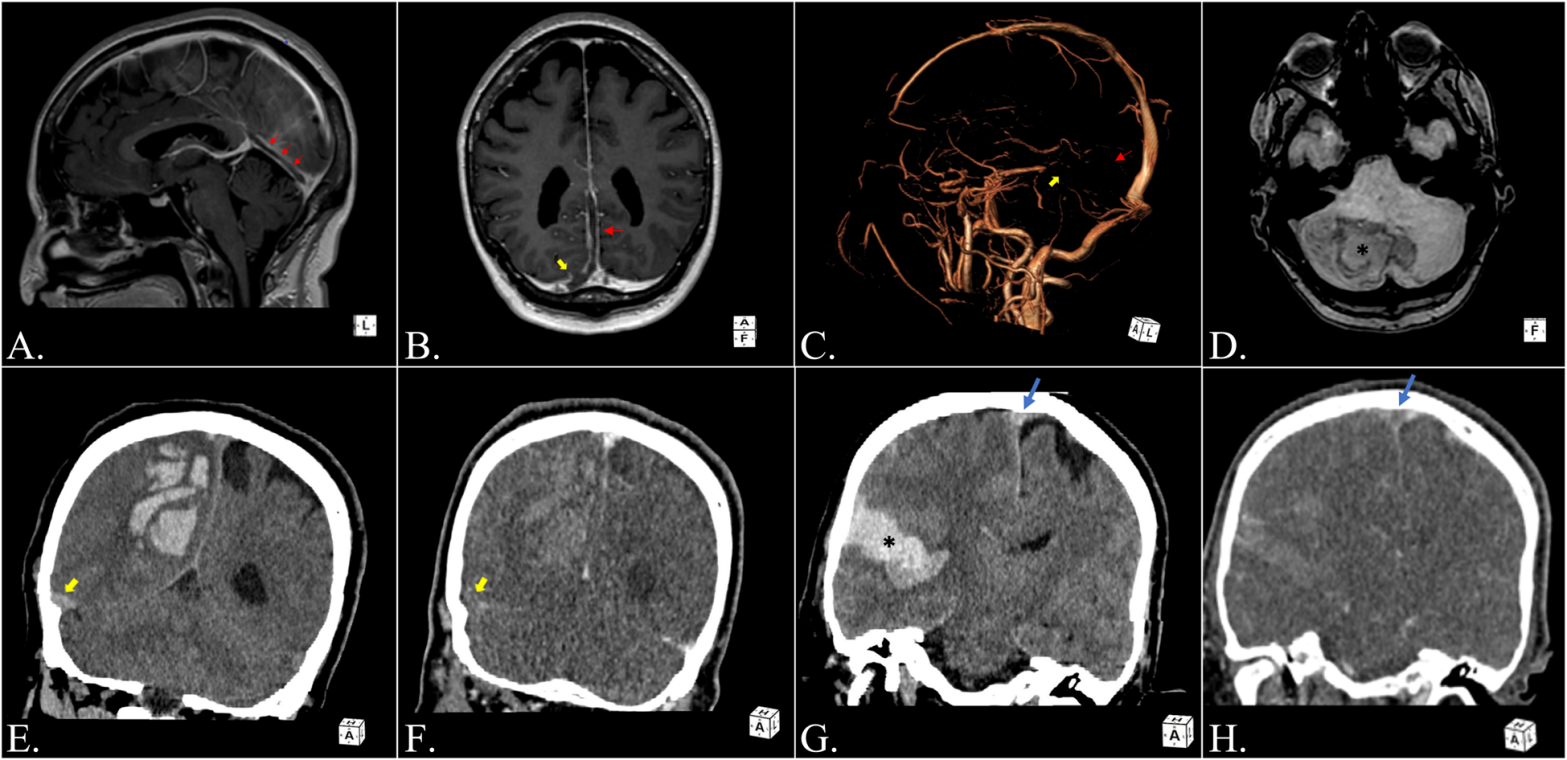
Neuroradiological findings. Patient 1: **A**, **D** magnetic resonance imaging (MRI) examination. **A**, **B** Sagittal and coronal contrast-enhanced T1-weigthed images depict SS (red arrows) and right TS (yellow arrows) extensive thrombosis with filling defect inside the vessels. **C** Phase-contrast MRI angiography (PCA) with volume rendering technique shows lack of venous flow in SS (red arrow) and right TS (yellow arrow). **D** Susceptibility-weighted (SWI) follow-up MRI examination shows acute bilateral cerebellar hemisphere hematoma, prevalent on the right side (asterisk). Patient 2: **E**, **H** brain computed tomography (CT scan examination) at clinical onset. **E** Coronal unenhanced CT scan depicts large right parietal hematoma and ipsilateral TS hyperdensity (“Dense clot Sign”), indicated by yellow arrow. **F** Coronal enhanced CT scan shows lack of opacization of thrombosed right TS (yellow arrow). **G** Coronal unenhanced CT scan depicts right temporal hematoma (asterisk) and “Dense clot Sign” at SSS (blue arrow). **H** Coronal enhanced CT scan shows triangular area of enhancement with a relatively low-attenuating center (“Empty delta Sign”) at thrombosed SSS (blue arrow). Straight sinus (SS), superior sagittal sinus (SSS), transverse sinus (TS)

Antibodies to platelet factor 4 (PF4) were positive in both patients, as previously described in some other reported cases of VITT [35]. Consistent with an assumed autoimmune mechanism, treatments for these VITT were anticoagulants different from heparin (fondaparinux), high dose intravenous immunoglobulins and steroids [36, 37]. Despite treatment, deterioration of neurological picture occurred, with refractory intracranial hypertension leading to brain death. Following patient will and thanks to organ perfusion/function maintenance, despite severe acute thrombotic events, successful organ donation was possible in both cases, with 6 months good outcomes in recipients.

As VITT is a potentially fatal event associated with vaccine against a much more devastating pandemic, it is imperative that science move forward illuminated by wisdom and prudence. High index of suspicion and prompt diagnosis are extremely important to ensure immediate hospitalization and therapy [36, 37], since CVST associated with either vaccines or COVID-19 seems to evolve much more rapidly and with a higher level of fatalities than CVST with different etiology [38].

Finally, if brain death occurs, careful organ donor management, including mitigating inflammatory response, can allow functional organs to be successfully transplanted to save other lives.

## Data Availability

Data sharing is not applicable to this article as no datasets were generated or analyzed during the current study.

## Abbreviations

VITT: Vaccine–induced immune thrombotic thrombocytopenia
SARS-CoV-2: Severe acute respiratory syndrome coronavirus 2
CVST: Cerebral venous sinus thrombosis
COVID-19: Coronavirus disease 2019
PF4: Platelet factor 4
HIT: Heparin-induced thrombocytopenia
aHIT: Autoimmune HIT
SS: Straight sinus
SSS: Superior sagittal sinus
TS: Transverse sinus
PCA: Phase-contrast angiography
SWI: Susceptibility-weighted
CT: Computed tomography
IVIG: Intravenous immunoglobulins
ACE2: Angiotensin-converting enzyme 2
